# Editorial

**Published:** 1871-04

**Authors:** 


### Editorial.

In the *American Journal of Obstetrics* for February, 1871, is an article on the introduction of sutures into the uterus after Cæsarian section, by Charles F. Rodenstein, M.D., of Westchester, N. Y., in which the following statement is made in regard to the rate of mortality of the mothers from that operation: "Lately I have had occasion to make the Cæsarian operation a subject of special study. I have collected the records of over four hundred cases, which have been operated on since the beginning of this century; of these about 43 per cent. have terminated fatally to the mother. I have examined especially the history of the fatal cases with reference to the causes of death. In a number of cases death was the result of intercurrent affections, which stood in no direct relationship to the operation itself; a certain number died from exhaustion; contrary to what might be expected, the proportion of deaths from hemorrhage and peritonitis is very small. Some, doubtless, died of mismanagement, either during the operation or in the after-treatment; but in the majority of cases the causes and manner of death is not yet fully understood." There is a liability of error in the conclusions drawn from any statistics or reports of cases that can be gathered together, arising from the fact that the successful cases are so much more generally reported than the fatal cases. Some good obstetrical authorities still claim that the Cæsarian operation is almost universally and necessarily fatal to the mother, and that its performance is never justifiable except as a last resort. Prof. Bedford, however, in his work on obstetrics, gives statistics showing a rate of mortality in this operation of 1 in $2\frac{1}{3}$ of the mothers, which is almost exactly the same rate as that deduced by the present writer. Prof. Bedford also says, further, that he is quite confident "if the alternative of Cæsarian section was more opportunely resorted to; if, in a word, the same principle of prudence should obtain in reference to it which we find to constitute the rule of action in all capital operations, the result would be vastly different, and I have no hesitation in saying that under these favorable circumstances, the Cæsarian operation would not only prove to be infinately less destructive to human life than craniotomy, but that it would soon take its rightful place as a just expedient in the lying-in chamber.

The evidence in demonstration of the soundness of this opinion seems to me to be entirely satisfactory; for, in addition to other proofs, we have the strong corroborative testimony furnished by those examples in which the Cæsarian operation has been performed several times on the same woman with success to both mother and child; and in these cases, it is fairly to be presumed that, at least, if not the first operation, the subsequent ones were undertaken opportunely before the strength of the mother had become exhausted by antecedent and protracted effort."

In reference to the introduction of sutures into the uterus in the operation, the writer in the *Journal of Obstetrics* goes on to say: "In examining the record of *post mortem* appearances, I was struck with the frequency of such statements as these: The 'uterus was found open'; 'the edges of the wound gaped'; 'the uterine incision did not close.'

In the histories of repeated Cæsarian section there is generally mention made of the condition of the previous uterine wound, and the manner in which it healed. And there also we find comparatively few cases in which the incision did fairly heal per prima or by cicatrization.

In some instances the edges of the wound were agglutinated to the abdominal walls; in other cases the gap was only closed by the peritoneal covering. And yet it is a universal observation that the womb does contract firmly, as a general thing, as soon as it is emptied of its contents, as well after a Cæsarian section as after any other delivery. And in the very accounts of the operations, which preceded the autopsies and the repeated Cæsarian sections, to which I refer, we read that the uterus did close before the operator proceeded to unite the abdominal wound. There must therefore be a period after the operation when the uterine wound is liable to open again.

It is not difficult to conceive how this may occur. We need only recollect that in delivery by Cæsarian section the os uteri has not been dilated to the same extent as in a birth per via naturales; that frequently immediately afterwards it is found closed; and that when the womb contracts upon the puerpural discharges, it is as easy for the uterine incision as for the natural outlet, to open for their exit.

The occurrence of this accident explains in part the mode of death in which many Cæsarian sections terminate. Many cases promised to do well at first, when sudden collapse—as sometimes happens in rupture of the uterus—ended the patient's life. Others die with symptoms of intestinal obstruction; and here an incarceration of a knuckle of intestines in the uterine wound may have taken place.

By closing the wound with sutures the danger of such unfortunate occurrences may be prevented. In a Cæsarian section, which I witnessed at Guy's Hospital in 1867, the wound did contract firmly after the extraction, and a speedy recovery of the patient was confidently expected; but in the transactions of the Obstetrical Society of London I find a report of her death from peritonitis, produced by discharges from the womb, which, as the autopsy proved, had opened again. And Braxton Hicks, who operated on that occasion, says: 'I cannot but help thinking that, had the uterine wound been closed in this case, many of the most serious complications would not have occurred.'

The seven cases in which sutures were introduced, were certainly among those of most unfavorable prognosis. Yet four were successful. In one fatal case, the sutures gave way, and the wound was found open; in another the sutures were purposely removed; and in the third, if the application of sutures did not save the life of the patient, it was at least the successful means of stopping an immense hemorrhage when all other measures had failed.

From the foregoing facts, I think the following conclusion may be legitimately drawn:

1st. The introduction of sutures is positively indicated when the uterine wound is not closed by the contractions of the womb.

2d. The closing of the uterine wound by sutures does not interfere with the success of the Cæsarian section.

3d. The application of uterine sutures after every Cæsarian section, will probably diminish the rate of mortality attending that operation.

---

Medical Legislation. — We think two or three bills have been presented to the Legislature of this State during its present session, purporting to "Regulate the Practice of Medicine and Surgery," in Illinois. The leading feature of these bills appears to be the establishment of some method by which it shall be ascertained that those who propose to enter upon the practice of medicine have received, at least, a respectable medical education, without attempting to discriminate between one pathy and another. One of these bills provides for the appointment of a board of censors in each county, by the probate or county judge, and the details of the other we have not seen. Twenty years since, we had occasion to examine the history of medical legislation in this country, with some degree of care, and the idea was strongly impressed upon us that there was little hope for permanently useful results from any legislation on the subject of medical practice. There is no doubt but the community sadly need protection from the enormous amount of imposition that is constantly being practised upon the afflicted, under the pretense of treating and curing disease.

But the appointment of boards of censors in every county, by judges who know no more about medicine or the qualifications of physicians, than any other intelligent members of the community, would be worse than useless.

To establish boards of censors in any way, and require such boards to recognize a college diploma as sufficient evidence of qualification to practice would defeat one of the most important objects in view. The number and variety of institutions called medical colleges, and the facility with which diplomas can be obtained from some of them, renders the mere possession of such a document no evidence of respectable attainments either in medicine or general science. For instance, we have in this city two regular medical colleges, one Homœopathic, one Eclectic, and what is represented on the sign-boards, as a *branch* of the Edinburgh University.

The latter is probably a simple agency for selling diplomas. The Eclectic establishment holds two college terms each year, of three or four months duration, and confers degrees at the end of each; thus enabling any man or woman to attend all the college terms required, and get the diploma in the short space of *eight* consecutive months. Similar facilities for obtaining the coveted diploma exist in all parts of the country. Hence we make no false accusation when we say that the possession of what purports to be a medical college diploma, is no positive evidence of a respectable medical education.

If the members of the Legislature desire to protect the people against the constant impositions practised on them by men and women under the name of *doctor*, let them enact a law providing, 1st, for the establishment of a single State Board of Examiners, whose duty it shall be to meet semi-annually and actually examine *all* persons who desire to enter upon the practice of medicine in this State, who do not hold a license from a similar board in another State, and to issue licenses to such only as they find properly qualified in all the departments of the science and art of medicine, whether they have college diplomas or not.

Such State Board of Examiners should be appointed by the Governor, on recommendation of the colleges and State societies, as follows: Every medical college organized under the laws of the State, having not less than *nine* active professors, an annual college term of not less than *five* months, and a curriculum embracing all the branches of study recommended by the Convention of College Delegates in Cincinnati, in May, 1867, should have the privilege of nominating one member of the Board, and every State medical society, organized for the advancement of the interests of medical science, and having an active membership, resident in the State, of not less than fifty practitioners, should have the privilege of nominating one member of the Board for every thirty of its resident members; the nominations to be made annually, and sent to the Governor on or before the 1st day of July in each year.

A majority of the Board so appointed should constitute a quorum for the transaction of business. They should keep a permanent record of their proceedings and examinations; and should admit no candidate to an examination for license, who did not present satisfactory evidence of having attained the age of 21 years; of having a good general education; of having studied medicine three full years, at least *one* of which should have been in some medical college with which was connected good facilities for hospital clinical instruction.

2d. After a given period, perhaps six months after the passage of the law and the appointment of the Board of Examiners, no person should be allowed to *commence* the practice of medicine in any of its departments in this State without a license from the State Board of Examiners, or from a board with similar powers in some other State. Every violation of this section of the law should subject the person committing the violation to a fine of not less than $50.00, nor more than $100.00, for each offence, to be recovered in any court in this State.

Such provisions properly carried out, would in the future secure to the community a respectably educated supply of physicians and surgeons, and effectually prevent the larger part of the impositions and quackery that are now so prevalent everywhere. A board of examiners constituted in the manner proposed, would also greatly encourage the maintenance of efficient State medical societies, and those colleges that actually possessed such organization and hospital connections as render them capable of giving full and thorough instruction in all departments of medicine; while they would equally discourage the multiplication of schools with incomplete organizations and inadequate facilities for instruction, and whose chief aim seems to be, to confer as many diplomas as possible, after the smallest requirements as to time of study and actual attainments.

The plan here proposed makes no discrimination in regard to *pathys* and *isms*. If a Homœopathic State Medical Society existed with the required number of resident members, it could nominate its pro rata number of candidates for appointment in the examining board. If a Homœopathic or Eclectic Medical College existed with the required organization and facilities for instruction, it could nominate its candidate for appointment as member of the board. Hence there could be no just opposition to such a law on the ground of monopoly or invidious distinction. It could be consistently supported by every individual who desired to secure to the community an educated and intelligent medical profession. If a majority of the board should be from the societies and colleges of the regular profession, it would be simply because such regular profession, in its schools and membership of its society, would greatly outnumber all the others.

We are aware that such a law, providing a State Board in which might be found some representatives of Homœopathic and Eclectic institutions, would meet, at least for a time, the severe censure of a large part of the regular profession. But after bestowing upon the subject much thought, we are fully satisfied of two things:

1st. That in this country, no law of any value regulating the practice of medicine can be maintained on the statue books of several States and rendered operative, which proposes to restrict the practice of medicine *exclusively* to those educated and licensed by the schools and societies of what is known as the regular profession. Consequently, the community must continue to support an army of pretenders, ignoramuses, etc., as at present, or the regular profession must consent to the establishment of such a *representative* board as will command the support of the more intelligent part of those who have become identified with some special system.

2d. That the practical working of such a board as here proposed, if the regular profession would give it a hearty support, would result in speedily breaking down the artificial and fictitious pretentions of all the special pathys and isms, that now delude the public with the idea of *new school* and old school, etc.

The bringing of all students of every name, before the same board, the exaction of the same amount of knowledge of the wide range of branches collateral to practical medicine proper, and the consequent comparison of true medical science with theoretical fancies, which would occur in the examinations, would inevitably break down the main props on which all the present pretended special systems of medicine rest. Such a board would in no respect necessitate any alteration of the present ethical rules of the profession. If a man obtaining his license choose to practice under the name of Homœopath or Eclectic there would be no more obligation to recognize him, in society or consultation, than at present. Every day men are found practising under these titles who have graduated at regular schools, yet no one regards the latter fact as imposing any obligation on the regular profession to recognize them as physicians in the ordinary relations of professional intercourse.

---

Chicago Medical College Commencement Exercises.—The exercises belonging to the public commencement of this institution were held on Monday and Tuesday, March 13th and 14th, in the lecture-room of the college.

At two o'clock P.M., on Monday, the class assembled in the lecture-room, and the candidates for graduation were subjected to a public examination on the branches belonging to the senior course.

Excellent theses were also read by E. J. Clark, J. T. Everett, and A. J. Smith.

On Tuesday, at 2½ P.M., the lecture-room was closely filled with an audience of gentlemen and ladies.

E. O. Haven, D.D., President of the Northwestern University presided, and, after music, opened the exercises with prayer.

The Dean of the Medical Faculty distributed the certificates of examination to the junior and middle-course students, accompanied by appropriate remarks. The Professor of Clinical Surgery delivered special certificates to such members of the senior class as had served as dressers or assistants in the hospital. The report of the committee appointed to examine the theses presented by the candidates for graduation, announced the award of the prize for the best thesis to Daniel Lichty; for the second best to Frank H. Davis, and made honorable mention of one by N. L. Kean.

The President of the University then conferred the degree of Doctor of Medicine on the following named gentlemen, accompanying the same with highly appropriate remarks:

David Henderson Alvis; Wilbur Parsons Buck; Amasa Franklin Chandler; Elbert Judson Clark; Harlan Page Cole; Frank Howard Davis; Joseph Warren Dysart; John Turner Everett; Josiah Wright Ghrist; Norman Louis Kean; Daniel Lichty; George Edwin Lord; Liston Homer Montgomery; Orrin William Moore; Anson Smith Munsell; John James Rousseau; Jacob Schneck; Andrew Jackson Smith; Theodore F. Stair; Alfred Swanson; J. Seymour Taylor; Daniel Ellsworth Thayer; Robert Thomas Williams; Henry Wilcox Westover.

*Ad Eundem Degrees.*—Chas. Badger, M.D.; O. W. Blanchard, M.D.; George Mathias Bell, M.D.; John G. Frank, M.D.

*Honorary Degrees.*—J. J. Clemmer; R. George English.

Daniel Lichty, in behalf of the graduating class, responded to the remarks of the President, in a most appropriate and elegantly-expressed address. After some excellent music by the club, R. N. Isham, Professor of Surgical Anatomy and Operations of Surgery, delivered the formal Valledictory Address, which was listened to by the class and audience with great pleasure and profit. Music and benediction closed the exercises of the twelfth annual commencement of this College.

An elegant social entertainment, in honor of the graduating class, was given in the evening, at the residence of Prof. E. Andrews.

The college term just closed has been a highly satisfactory and prosperous one. While the classes in most of the medical colleges throughout the country, have diminished in number as compared with former years, there has been a decided increase here, especially in the junior and middle divisions of the class. Such a result in connection with the long lecture term; the graded classes; the annual examinations; and the ample hospital clinical facilities, is particularly gratifying to those who earnestly desire to see the whole system of medical education in this country placed on a broader basis, and reduced to a more systematic order.

---

American Medical Association.—Arrangements have been completed with the Northwestern and Union Pacific Railroads to carry members of the profession who desire to attend the annual meeting of the American Medical Association, on the 2d day of May, 1871, in SanFrancisco, from Chicago to Omaha and back for $24.00, and from Omaha to SanFrancisco and back for $125.00, making $149.00 for the round trip from this place.

Tickets can be purchased at any time after the 15th of April, and will be good for sixty days from date of purchase.

Physicians can take members of their own families with them at the same rates. Tickets from Chicago to Omaha and back can be purchased at the ticket-office, 125 Lake Street, *only.* From Omaha to SanFrancisco and back at the office in Omaha. But all applicants for commutation tickets will be required to show certificates that they are to be recognized as actual members of the SanFrancisco meeting. Consequently, all in the

Northwest who intend to go, had better send to me, without delay, their names and address, with the names of such members of their families as they desire to take with them.

N. S. DAVIS,
Local Committee on Railroads,
797 Wabash Avenue, Chicago, Ill.

---

ILLINOIS STATE MEDICAL SOCIETY.—The next annual meeting of this Society will be held in Peoria, on the *third Tuesday* in May. The meeting will be one of much interest, and we hope every part of the State will be represented. A full statement of the programme will doubtless be furnished by the Society in time for our next issue.

---

SUMMER COURSE OF MEDICAL INSTRUCTION.—The regular summer course of instruction in the Chicago Medical College, Medical Department of Northwestern University, will commence on Monday, April 3, 1871. It consists of daily clinical instruction in the hospital and dispensary, and two lectures per day by members of the faculty. It is free to all regular matriculates of the College.

---

THE Executive Committee of the Alumni Association of the Medical Department of the University of the City of New York propose the publication, at the earliest possible date, of a complete catalogue of the graduates from that institution since its foundation. The records of the Faculty having been destroyed in the burning of the college building some years ago, this project is one that should be seconded by every one of the alumni, of whom between two and three thousand are scattered throughout the United States. It is earnestly requested that each of these will without delay forward for enrolment his full name and post office address, with his professional history, including date of graduation, posts of honor and trust held, etc., and also any information which he may possess concerning former classmates who have since died or retired from practice. Communications should be addressed to the Secretary, Charles Inslee Pardee, M.D., 72 West 35th Street, New York.

POSTPONEMENT.—The second-half of the paper on "Alcohol and Tobacco" is deferred until the May number.

---

REMOVAL.—The old and well-known drug store of Bliss & Sharp, formerly J. H. Reed & Co., so long kept at 144 Lake Street, has been removed to 105 State Street; where their friends will find them with their usual full assortment of Drugs, Chemicals and Surgical Instruments. In their new store one entire floor is devoted to the display of Surgical Instruments, of which they have the largest stock in the West.

---

AN OPERATION FOR THE CURE OF OBSTRUCTIVE DYSMENORRHŒA.—Prof. T. G. Thomas (*Medical and Surgical Reporter*) in a case of this affection, the obstruction being at the external os which barely admitted a fine probe, removed with a bistoury a small ring of cervical tissue from about the os. He speaks of the operation as devoid of danger.

---

DIGITALIS LEAVES IN ORCHITIS.—Dr. Besnier advises a concentrated infusion of the above leaves applied to the scrotum in orchitis and hydrocele. Dr. Given recommends elsewhere in this journal a somewhat similar treatment of orchitis.—*American Practitioner.*

---

CHRONIC CATARRH.—The tincture of aconite in five-drop doses every four hours has cured this troublesome symptom when the ordinary remedies have failed.—*Medical Archives.*

---

TO DISGUISE THE TASTE OF COD-LIVER OIL.—Dr. A. S. Hudson states (*Pacific Medical and Surgical Journal*) that tr. of gum guaiacum half an ounce, and essence of gaultheria one drachm, added to one pint of cod-liver oil, wholly covers the taste of the oil.

---

DEATH FROM CHLOROFORM.—Dr. Hughes Bennett stated, at a late meeting of the British Association, that "he knew of one very sad case that had happened in Edinburgh. A young and beautiful lady, daughter of a barrister, in perfect health, went to a dentist's house one morning, and had a tooth extracted. Five minutes afterwards she was dead. That was only one of many similar cases that had occurred, but had never been published."—*British Medicvl Journal.*

### Mortality for the Month of February, 1871.

| | | | | | |
|---|---|---|---|---|---|
| Accidents, drowned | 1 | Diarrhœa, chronic | 2 | Liver, abscess of | 1 |
| " brain concussion | 2 | " following child- | | Lungs, congestion of | 8 |
| " burns | 1 | birth | 1 | " hemorrhage of | 1 |
| " crushed | 1 | " & complications | 1 | " paralysis of | 1 |
| " fracture of skull | 1 | Diphtheria | 10 | Manslaughter | 1 |
| " burned by gasoline | 1 | Dropsy general | 3 | Measles | 5 |
| " railroad | 4 | Dysentery | 2 | " and complications | 1 |
| Anæmia | 1 | Enteritis | 9 | Metroperitonitis | 1 |
| Anasarca | 1 | Epilepsy | 1 | Meningitis | 4 |
| Apoplexy | 5 | Erysipelas | 5 | " cerebro spinal | 4 |
| Asphyxia | 1 | Exposure | 1 | " tubercular | 3 |
| Asthma | 1 | Fever intermittent | 1 | Old age | 11 |
| Aorta, aneurism of | 1 | " congestive | 1 | Peritonitis | 5 |
| Atrophy | 1 | " puerperal, | 2 | Peritonitis puerpural | 1 |
| Bowels, congestion of | 1 | " remittent | 1 | Pleurisy, chronic | 1 |
| Brain, congestion of | 6 | " scarlet | 3 | Pneumonia | 35 |
| " disease of | 2 | " & complications | 3 | " broncho | 4 |
| " inflammation of | 4 | " malignant | 3 | " and complications | 5 |
| " softening of | 1 | " typhoid | 7 | " typhoid | 3 |
| Bronchitis | 9 | Gangrene | 1 | Pyæmia | 1 |
| " capillary | 6 | Heart disease | 5 | Rheumatism | 1 |
| Cancer of breast | 1 | " dropsy of | 2 | " inflammatory | 1 |
| " of rectum | 1 | " hypertrophy of | 1 | Small-pox | 2 |
| " of stomach | 4 | " fatty degeneration | 1 | Sorethroat | 1 |
| " of uterus | 1 | " organic disease of | 4 | Spine, disease of | 1 |
| Child-birth | 1 | " valvular disease | 3 | Stomach, perforation of | 1 |
| Consumption | 46 | Hip-disease | 1 | " ulcer of | 1 |
| Convulsions | 41 | Hepatitis, | 2 | Stricture and absorb- | |
| " epileptic | 1 | Hydrocephalus | 4 | tion of urine | 1 |
| Croup | 18 | " acute | 2 | Tabes mesenterica | 8 |
| " diphtheritic | 3 | " chronic | 1 | Teething and compli- | |
| " membraneous | 5 | Insanity & dysentery | 1 | cations | 1 |
| Cyanosis | 1 | Inanition | 12 | Uterus, hemorrhage of | 1 |
| Debility, general | 2 | Jaundice | 1 | Whooping-cough | 2 |
| " following child- | | Kidneys, Bright's dis- | | " & complications | 1 |
| birth | 1 | ease of | 6 | | —— |
| Deficient development | 1 | Laryngitis | 1 | Total | 400 |

Premature births 10 | Still births 59 | Total 69

### AGES.

| | | | | | |
|---|---|---|---|---|---|
| Under 1 | 128 | 10 to 20 | 15 | 70 to 80 | 8 |
| 1 to 2 | 39 | 20 to 30 | 42 | 80 to 90 | 5 |
| 2 to 3 | 22 | 30 to 40 | 44 | 90 to 100 | 1 |
| 3 to 4 | 9 | 40 to 50 | 21 | | —— |
| 4 to 5 | 13 | 50 to 60 | 28 | Total, | 400 |
| 5 to 10 | 14 | 60 to 70 | 11 | | |

| | | | | | |
|---|---|---|---|---|---|
| Males, | 218 | Females, | 182 | Total, | 400 |
| Single, | 279 | Married | 121 | Total, | 400 |
| White, | 395 | Colored, | 5 | Total, | 400 |

### COMPARISON.

Deaths in Feb., 1871, 400 | Deaths in Feb., 1870, 421 | Decrease, 21

Deaths in Jan., 1871, 415 | Decrease, 51

NATIVITY.

| | | | | | |
|---|---|---|---|---|---|
| Atlantic Ocean | | Denmark | | New Brunswick | 1 |
| Austria | 1 | England | 10 | Norway | 7 |
| Bohemia | 4 | France | | Switzerland | 2 |
| Canada | 5 | Germany | 43 | Scotland, | 5 |
| Chicago, Native | 69 | Holland | 2 | Sweden | 5 |
| Chicago, Foreign | 135 | Ireland | 38 | Wales | |
| U. S., other parts | 72 | Italy | | Unknown | 1 |
| | | | | Total, | 400 |

MORTALITY BY WARDS FOR THE MONTH.

| Wards. | Mortality. | Wards. | Mortality |
|---|---|---|---|
| 1 | 5 | 18 | 18 |
| 2 | 12 | 19 | 11 |
| 3 | 16 | 20 | 21 |
| 4 | 6 | Accidents | 11 |
| 5 | 21 | County Hospital | 7 |
| 6 | 24 | Home for Friendless | 4 |
| 7 | 14 | Hospital Alexian Bros. | 3 |
| 8 | 36 | Foundling Home | 1 |
| 9 | 33 | Manslaughter | 1 |
| 10 | 15 | Protestant Orphan Asylum | 1 |
| 11 | 14 | St. Luke's Hospital | 1 |
| 12 | 15 | Woman's Home | 1 |
| 13 | 12 | Marine Hospital | 1 |
| 14 | 8 | Scammon Hospital | 1 |
| 15 | 42 | St. Joseph Orphan Asylum | 7 |
| 16 | 17 | | |
| 17 | 21 | Total, | 400 |

Mean Thermometer, 30½°, Rain, 2,220 inches, Deaths daily, 10⅗.

---

Money Receipts from February 25 to March 25.—Dr. J. S. Jenks, Plano, Ill., $3.00; Dr. J. W. Boggis, Coon Rapids, Iowa, 3.00; Dr. Thomas Hamill, Olathe, Kan., 3.00; Dr. J. P. Conont, Prairie du Chien, Wis., 10.00; Dr. C. M. Robertson, Tallula, Ill., 5.00; Dr. G. S. Thomas, City, 6.00; Dr. J. H. Judson, Polo, Ill., 3.00; Dr. J. B. Carr, Munroe, Iowa, 3.00; Dr. H. H. Sloan, City, 3.00; Dr. C. Goodbroke, Clinton, Ill., 10.00; Dr. R. L. Wolston, Paris, Ill.' 6.00; Dr. J. M. Laughlin, Montville, Minn., 3.00; Dr. H. B. Wilcox, Three Oaks, Mich., 8.00; Dr. Bond, Johnstown, Wis., 9.00; Dr, J. McMillan, Biggsville, Ill., 3.00; Dr. J. T. Everett, City, 3.00; Dr. W. H. Searles, Warsaw, Wis., 3.00; Dr. O. L. McArthur, Rockford, Ill., 18.00; Dr. H. Rodbaugh, Diana, Ill., 3.00.

---

In the February number of the *New York Medical Journal* there is reported one case of asphyxia from drowning, and two of phthisis pulmonalis treated by the use of oxygen gas, at the Long Island College Hospital. The writer remarks in regard to these cases:

"The first case is of interest as showing the effects of oxygen gas as a resuscitating agent in cases of asphyxia, and is, as far as I have seen, the only reported case in which it has been used in asphyxia from drowning. But for its use in the foregoing case, there is not much probability that the patient would have rallied. It will be seen that the patient, from an apparently moribund condition, began immediately to improve on the use of the oxygen gas, until reaction was complete, and, but for the supervening pneumonia, there is no evident reason why complete recovery would not have taken place. In the two remaining cases, the first effect of the oxygen, as will be seen, was to increase the pulse and temperature 'slightly,' for the first three or four days; after that the pulse steadily decreased to its normal standard. The first favorable symptom manifested was an 'increase of appetite'; the assimilative powers of the gas seemed very marked, promoting digestion from the beginning; this was especially shown in the case of McLavy, whose diarrhœa almost entirely disappeared during the second week after commencing the inhalations; gain in flesh following immediately upon the improved appetite and declining diarrhœa. It is a noticeable fact with regard to taking the cod-liver oil, that it disagreed with the patients as long as they were *confined to the house*, and produced nausea and sometimes vomiting, to such an extent that it was necessary to discontinue it for several days; the oxygen, however, supplying, to a certain extent, fresh outdoor air, its exhibition was attended with a steady decrease of the unpleasant symptoms caused by the oil, and when the patients were enabled to spend most of the day out-of-doors, in addition to taking their daily supply of the gas, the effect of the oil was most beneficial. It will be observed that the *weight* is made the criterion of improvement, the increase being steady in both cases, but much more rapid in the case of McLavy than of Yorgo; and it may be well to remark here that the former gave no family history of tuberculosis, being a genuine case of Niemeyer's 'phthisis pulmonalis,' whereas the latter inherited tubercles, having lost several of his immediate family with this disease. The patients very readily learn to inhale the gas after one or two trials. It is taken in a recumbent position, an ordinary respiration alternating with one of the gas. The gas is made in the laboratory of the college adjoining the hospital, under the direction of the house-staff, and at a small expense—averaging only from one and a-half to two cents per gallon."

---

TREATMENT OF DELIRIUM TREMENS.—Concerning this vexed question, Dr. Murchison says (*London Lancet*), in regard to—

1. *Alcohol.* He allows none, except in cases where there is evidence of fatty heart, or an intermitting pulse, or some special complication, as phthisis. He has never seen any bad consequences from suddenly and completely cutting off the supply of alcohol.

2. *Food.* Mild cases are often cured by good nutriment and abstaining from stimulants; but sleep will not follow this practice in severe cases, while in not a few bad cases there is congestion of the liver and stomach, and food of all kinds is rejected.

3. *Opium.* In many cases opium acts like a charm in speedily putting an end to the disease; while in others it fails entirely in inducing sleep, or may aggravate the symptoms, or even cause convulsions and coma. Is there no explanation of this difference? Is it impossible to say when opium is likely to succeed or not? or must we, from being uncertain of the result, abjure the use of it altogether? He thinks an explanation of the difference is to be found in the state of the kidneys, as indicated by the characters of the urine. Whenever the urine contains albumen as the result of recent congestion, or old disease of the kidneys, opium is almost sure to fail, and even prove injurious; and accordingly it is a good rule never to give opium until an opportunity has been offered to test the urine. But when the urine has been ascertained to be free from albumen, opium may be given without fear, and usually with the best results. It is best to commence with a full dose, and give a smaller dose every three hours afterward until sleep ensues. When the skin is dry, or the patient much excited, combine the opium with antimony in the manner recommended by the late Dr. Graves.

4. *Digitalis* is of undoubted power in the treatment of delirium tremens, and is particularly indicated in cases where the urine is scanty or contains albumen, or where the patient is very excited. He has known it to act most beneficially in cases where opium had failed. The large flow of urine following its use makes it probable that it assists in the removal of deleterious matters from the blood. From fifteen to thirty minims of the tincture may be given with or without carbonate of ammonia, every four hours.

5. *Bromide of Potassium.* In severe cases he has not found it alone of much service in securing sleep, although it has seemed to act beneficially in moderating active delirium or mental excitement.

6. *Hydrate of Chloral* is a remedy for inducing sleep which is particularly applicable to those cases where opium is contra-indicated. It does not, like opium, interfere with elimination by the kidneys. One caution is necessary with regard to it. Not only in delirium tremens, but in other diseases, the first action of the chloral (like that of an insufficient dose of chloroform) is exciting rather than sedative. You must not on that account infer that it is acting injuriously, for a second dose will often produce the desired sleep. The best way to give it is in doses of half a drachm every two or three hours until sleep results.

---

CAUTERIZATION IN DIPHTHERIA.—In the *48th Versammlung Deutsche Naturforscher und Aerzte*, Dr. Schuller stated that he had entirely abandoned cauterization of the pharynx, larynx, or conjunctiva in diphtheria. In numerous cases he had, as a crucial experiment, cauterized only one side of the fauces, and he had always been led to the same conclusions:

1st. That the membrane remained attached longer on the side which he had cauterized than the other.

2d. That even the most energetic application of nitrate of silver failed to arrest the reproduction or to prevent the extension of the membrane.

3d. In some cases serious tumefaction and inflammation of the cervical lymphatics followed the application of the caustic.

In these views he was supported by Ebert, Stiebel, Cohen, Rinecker, and others, who direct the use of small pieces of ice to be constantly allowed to melt in the mouth, and employ a gargle of potass. chlor., alcohol, potass. permang., carbolic acid, etc.—*The Medical Times.*

---

SYMPATHETIC NERVOUS TROUBLES FROM THE PRESENCE OF A TENIA.—Two cases, mentioned by Dr. Maurin, (in *Sud Medical* No. 5, 1870, and *Lyon Medical*, April 24, 1870), are interesting in this, that they might have given rise to a diagnosis of troubles of the nervous centres.

The first patient was a clerk, aged 42, who for fourteen months had experienced more and more serious illnesses. At first he suffered from dull pains in the epigastrium; these pains, after a while, took on the character of an intense gastralgia, with inexplicable remissions and exacerbations.

A few months afterward he had some vertigo complicating the gastric pain. Finally, for eighteen months past, the gastralgia has yielded, but has been followed by a continued sensation of vertigo, a fixed pain in the nape of the neck and between the shoulders; sensation of falling forward when the patient walks up or down any incline; sensation of a soft body, like a cushion, under the feet during the walk; fear of an imminent death; sudden inclination to commit suicide.

The second patient was a physician who experienced all the symptoms of a cerebral congestion; cephalalgia, troubles of the intellect, incomplete amnesia, impediment of speech; the patient, being gouty, had fear of a metastasis; but a few articulations of tenia having been passed, the diagnosis was made, and, as in the preceding case, a dose of kousso, by producing the expulsion of a tenia, caused at the same time all his symptoms to disappear.—*New York Mecical Journal.*

---

THORACENTESIS.—Dr. James Cuming, Belfast, Ireland, gives (*Dublin Quarterly*) the following practical rules laid down by Bartels regarding the selection of cases in which thoracentesis is to be performed: "In all cases of simple serous effusion, accompanied by signs of displacement, the operation is requisite if the physical signs show that absorption has not commenced within a moderate time. It is not advisable to operate as long as febrile symptoms are present, unless there be urgent symptoms, such as distinct and considerable embarrassment of the circulation or of the respiration. The entrance of air into the pleural cavity is to be carefully prevented in cases of serous effusion. Purulent effusions are best treated by the establishment of a large fistulous opening, which permits a continuous discharge of the thoracic contents. If these effusions are removed by the trocar they rapidly accumulate afresh and exhaust the patient. If on puncturing the chest an effusion which had been regarded as serous is found to be purulent, it is advisable to remove the trocar and make a pretty large opening at once. The effusion is almost invariably purulent if pleurisy has occurred in connection with pyæmia, puerperal fever, and the like; if a febrile condition continues without any other cause after the effusion has ceased to increase; and is certainly purulent if œdema of the subcutaneous cellular tissue exists on the affected side. If pneumo-thorax coexist with purulent effusion, the operation is indispensible to prevent the contamination of the system by septic fluids. To prevent septic infection it is necessary to cleanse the pleural sac daily, either by injections of water or of a weak solution of common salt, or by insufflation of air. Opening the cavity of the thorax by means of a bistoury is reserved for those cases in which a permanent fistulous opening is required." Dr. C. himself thinks the pneumatic aspirator

(vide *American Practitioner* for August, 1870), possesses advantages over any other instrument for this operation.—*American Practitioner.*

---

Perforating Ulcer of the Bladder.—Mr. Lawson Tait narrates (*Lancet*) two instances of *chronic perforating ulcer* of the bladder in the female, which were under the charge of Sir James Y. Simpson, and which were cured by making a urethro-vesico-vaginal fistula. Rokitansky refers to this form of disease under the name of limited perforating ulcer. In anatomical characters, as well as in semeiology, the ulcer closely resembles the perforating ulcer of the stomach, and like it may sometimes prove fatal from perforation occurring and causing peritonitis. The symptoms are intense pain about the neck of the bladder, greatly aggravated by micturition; a few minutes of comparative ease after the bladder is emptied, but the pain steadily increasing with its distension; the urine slightly alkaline, with a small quantity of pus, and in one case having a trace of albumen.

The operation is performed by introducing a grooved staff in the urethra, and slitting up the posterior fourth of the canal, and about an inch of the posterior wall of the bladder. There is no difficulty in getting the fistula to close after the ulcer is healed; the difficulty is to get it to remain open long enough. The object aimed at in this treatment is to put the bladder in a state of complete physiological rest.

[Making a vesico-vaginal fistula in cases of obstinate cystitis is a practice that we believe to have been original with our distinguished countryman, Dr. Thos. A. Emmett. Mr. Tait does not give the date of Prof. Simpson's first operation, nor do we know that of Dr. Emmet's; so that the question of priority is uncertain.—*Eds. American Practitioner.*]

---

Rules in Operating on the Tongue.—Dr. Gurdon Buck gives the following directions (*Medical Record*) to be observed in operating for hypertrophy of this organ: A strong ligature traversing the tongue far back controls it and prevents retraction. Patient's head, resting against assistant's breast, is fixed by a hand on each side, and the forefingers retracting the angles of the mouth. The two other assistants, one on each side, draw the tongue forward by the ligature. The operator grasps the tongue laterally with the vulsellum, thus diminishing its breadth and increasing its thickness. He then with a long-bladed straight knife transfixes it far back within the mouth, midway between its upper and lower surfaces, and cutting forward and downward forms the inferior flap. The superior flap is formed in the reverse direction. Applying the middle of the edge of the knife to the upper surface of the tongue, opposite to the end of the flap just formed, so that they shall be of equal length, he cuts backward and downward, and terminates this section where the first began. The tongue during the action of the knife being compressed laterally, the extremities of the flaps will be rounded and will correspond to each other. The tongue being under perfect control by means of the ligature traversing it, the bleeding arteries may be secured with almost the same facility as after the amputation of a limb. The flaps are to be approximated and secured with sutures in the usual manner. An important advantage of this method is that the frænum linguæ, not being involved in the section of the tongue, continues to perform its office undisturbed.—*American Practitioner.*

---

Spontaneous Origin of Enteric Fever.—Dr. Charles E. Pryor, in the *London Lancet*, for December, undertakes to prove, by an ingenious and able argument, that enteric, or typhoid fever, is strictly contagious, and belongs, in point of fact, to the contagious exanthems. If he were to spend a few years in California, he might elaborate an argument more plausible and conclusive, to prove the very opposite, based on the history of the disease on this coast. We append his several conclusions, some of which have our hearty assent:

1. Spontaneous generation of plants and animals is a figment which is constantly receding as means of observation extend and improve.
2. Spontaneous generation of parasitical diseases is a figment.
3. The exanthemata may be a low form of fungoid life.
4. Smallpox, a contagious exanthem, is proved by indisputable negative testimony to be incapable of spontaneous generation.
5. Several other contageous exanthemata can not originate spontaneously.
6. Enteric fever is a contagious exanthem.
7. Febrile diseases of local origin are not contagious.
8. Experiment* gives strong evidence against the spontaneous origin of enteric fever.
9. Observation, as usually conducted, is a treacherous and insufficient test of the origin of febrile diseases.—*Pacific Medical Journal.*

* That is the only kind of experiment which is permissible.

Albuminuric Retinitis.—Dr. Argyll Robertson, on this subject, read (*Edinburgh Medical Journal*) a paper before the Edinburgh Medico-Chirurgical Society. He states this to be a fatty degeneration consequent upon inflammatory exudation; it was a late and not an early symptom of albuminuria. The amaurosis of pregnant women might depend on simple retinitis, on atrophy, or on anæmia of the optic nerve; or it might be associated with albuminuria, and then its chief importance is in reference to prognosis. According to Dr. Grainger Stewart this form of retinitis is generally found associated with contracting kidney, rarely with waxy degeneration. Prof. Bennett spoke of the important part which fatty degeneration played in pregnant and parturient women, the mode in which hypertrophied uterine tissue was removed, and also that by which the mammary secretion was prepared and elaborated. It was not surprising that interference with excretory functions should lead to fatty transformations of tissue.—*American Practitioner.*

---

Intracranial Abscess Cured by Trephining.—Prof. N. R. Smith narrates (*Baltimore Medical Journal*) a case of this kind. The subject had received, twenty years before, an injury upon the left parietal bone from a sharp stone. Inflammation of the pericranium followed, resulting in suppuration and detachment; then necrosis and discharge of a small sequestrum, involving both tables. A permanent fistulous opening remained. His general health remained good; occasional vertigo and pain in the head, especially when the discharge was impeded. Upon introducing a probe it passed fully two inches and a-half before reaching the membrane of the brain, and an abscess was traced out almost co-extensive with the parietal bone. The bone was thickened and of ivory hardness. A trephine seven-eighths of an inch was used; on the removal of the button three ounces of fetid pus escaped. No cerebral disturbance or other morbid phenomenon occurred. The dura-mater gradually rose to its normal place, and the cavity became obliterated. A year after the patient remained quite well.—*American Practioner.*

---

Chloroform in Labor.—Dr. Graily Hewitt, in the Obstetrical Society of London (*Medical Press and Circular*) uttered the following language: "We have come—some of us, at all events—to recognize the fact that chloroform has a tendency to make labor 'lingering;' that it sometimes enfeebles the uterus, and may thus cause hemorrhage. This tendency it is proposed to do away with, by diluting the chloroform by mixture of alcohol or other vapors, or by accurate mixture with air. Dr. Sansom has pointed out the great liability to the inhalation of poisonously high percentages of chloroform at high temperatures, unless proper care be exercised. Mr. Ellis has given us new inhalers for effecting such mixtures. The general conclusion I take to be, that, in ordinary midwifery practice, the anesthetic should be diluted, that it should not be given to produce the full effect, and that in all cases, rather excessive precautions against hemorrhage are required when chloroform is given."—*Pacific Medical Journal.*

---

Gonorrhœa Produceable by Chancre.—The doctrine of Hunter on this subject, which had been regarded as entirely overturned by the experiments of Ricord, are about to be reestablished. Prof. Hammond, of New York, after investigating the question, comes to the following conclusions, which correspond with those of some other recent inquirers:

"1st. That the virus of an infecting chancre, when deposited on a secreting mucous surface upon which there is no solution of continuity, may give rise to gonorrhœa unattended by chancre, but which is syphilitic in its character, and capable of producing constitutional disease.

"2d. The matter of such a gonorrhœa is capable of causing an infecting chancre, either by natural or artificial inoculation, which chancre is followed by constitutional syphilis."—*Pacific Medical Journal.*

---

Death from Inhalation of Ether.—At last a veritable case of directly fatal result from ether inhalation comes to light. It occurred in Boston, in the person of a man who had received a bullet wound in the knee, and who was etherized for the purpose of amputation. He suddenly ceased to breathe during the operation. In nearly every instance of death hitherto imputed to ether, hours, if not days, have elapsed before the fatal result. But the present case is different, resembling in this respect the action of chloroform. The particulars are given in the *Boston Medical and Surgical Journal*, of December 8, 1870.—*Pacific Medical Journal.*

---

Small Pox in London.—A severe epidemic of small-pox is now raging in London. The deaths have risen from an average of forty-two in the three preceeding weeks to sixty for the week ending December 3d, the highest number returned in any week since June, 1863.

